# Knowledge, Attitudes, and Practices Related to Treatment and Prevention of Cholera, Haiti, 2010

**DOI:** 10.3201/eid1711.110818

**Published:** 2011-11

**Authors:** Valery E.M. Beau De Rochars, Julie Tipret, Molly Patrick, Lara Jacobson, Kamil E. Barbour, David Berendes, Diana Bensyl, Cathie Frazier, Jean W. Domercant, Roodly Archer, Thierry Roels, Jordan W. Tappero, Thomas Handzel

**Affiliations:** Centers for Disease Control and Prevention, Atlanta, Georgia, USA (V.E.M. Beau De Rochars, M. Patrick, L. Jacobson, K.E. Barbour, D. Berendes, D. Bensyl, C. Frazier, R. Archer, T. Roels, J.W. Tappero, T. Handzel); Groupe de Recherche et d’Echanges Technologiques, Port-au-Prince, Haiti (J. Tipret);; Centers for Disease Control and Prevention Port-au-Prince (J.W. Domercant)

**Keywords:** Cholera, knowledge, attitudes, practices, outbreak, Haiti

## Abstract

In response to the recent cholera outbreak, a public health response targeted high-risk communities, including resource-poor communities in Port-au-Prince, Haiti. A survey covering knowledge and practices indicated that hygiene messages were received and induced behavior change, specifically related to water treatment practices. Self-reported household water treatment increased from 30.3% to 73.9%.

Haiti had not experienced an outbreak of cholera for more than half a century. This changed in October 2010 when a large outbreak occurred in Artibonite Department and quickly spread to the remaining departments of Haiti, including the city of Port-au-Prince (*1*). Given the prevalence of known risk factors for explosive spread of the disease (e.g., low socioeconomic status, high population density), an emergency public health response was initiated. With crowded conditions and limited access to safe water and sanitation, persons living in the capital of Port-au-Prince were especially vulnerable to acquiring cholera (*2*–*5*). This risk was exacerbated by the January 12, 2010, earthquake, which led to >1.5 million persons seeking shelter and services at internally displaced persons settlements in and around the capital (*6*). The first cases were confirmed in Port-au-Prince on November 7, 2010. As of August 8, 2011, Port-au-Prince had reported 112,464 cholera cases and 760 deaths (*6*).

In response to the cholera outbreak, the Haiti government and partner agencies initiated emergency public health response activities aimed at treating suspected cholera cases and preventing new ones. Response activities included mass media cholera campaigns through radio and hygiene promotion activities by community health workers, distribution of water purification tablets and soap, and limited distribution of oral rehydration solution (ORS) sachets. Prevention efforts focused on internally displaced person settlements in Port-au-Prince and the poorer neighborhoods of the city where information regarding cholera knowledge, dissemination of cholera information, and distribution of treatment and prevention supplies was limited.

## The Study

During December 6–7 and 14–16, 2010, we conducted a survey to assess the effectiveness of interventions implemented to prevent the spread of cholera and to improve specific response activities in these neighborhoods. Because this investigation was a public health response to an emergency, the Centers for Disease Control and Prevention determined that institutional review board review was not necessary. Informed consent was obtained from all participants.

The survey collected cross-sectional data on household demographics, communications preferences, knowledge of cholera transmission and prevention, water sources and treatment, and hygiene practices. Samples of stored water in the home were tested for chlorine residue by using the Hach Free Chlorine Test (Hach Co., Loveland, CO, USA) to provide an objective measure of water treatment. Microbiological testing for *Escherichia coli* by using IDEXX Quanti-Tray/2000 (IDEXX Laboratories, Inc., Westbrook, ME, USA) was also conducted on source water. Sampling weights according to the population size were used to improve the overall representativeness of results.

A household questionnaire (Technical Appendix) was pilot tested and administered to 405 households from 27 clusters from resources-limited areas of Cité Soleil, Delmas, Carrefour, and Pétion-Ville (Figure). Clusters were randomly selected by using population proportional to size sampling, with the exception of Cité Soleil, which was undersampled to provide more geographic representation in the sample. In each of the 27 selected clusters, 15 households were selected randomly along a radius from the edge to the center of the cluster.

**Figure:**
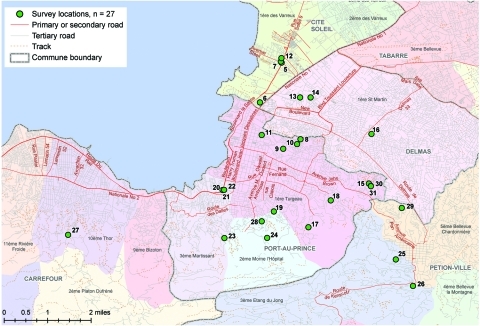
Selected clusters for the knowledge, attitudes, and practices related to treatment and prevention of cholera survey administered during December 6−7 and 14−16, 2010, Port-au-Prince, Haiti.

Persons interviewed were primarily female heads of households (81%). Average household size was 5 persons, and median age of respondents was 35 years (range 17–80 years). Most respondents had access to a cellular telephone (88.1%), radio (67.1%), and television (66.3%). The preferred forms of communication for receiving cholera messages were television (71.1%), radio (68.8%), and trucks with megaphones (44.0%). Knowledge of common signs of cholera was high; the 2 most common signs described were diarrhea (89.1%) and vomiting (83.4%). Respondents also showed high knowledge of transmission modes; 71.9% indicated consumption of contaminated water and 61.4% indicated consumption of contaminated food. The most common prevention method cited was handwashing (86.0%).

 Before the outbreak, the most common drinking water sources were piped water to tap stands and public kiosks (Table 1). These water sources were chlorinated irregularly, and only 6.2% of respondents believed that drinking water from the piped supplies was safe. Microbiological testing of 11 unchlorinated piped water sources indicated that 7 were positive for an indicator of fecal contamination (*E. coli*). Collection of tap water decreased during the cholera outbreak, whereas collection of drinking water from private kiosks nearly doubled (47.6%). Public health messages on the health benefits of water treatment showed diffusion in these neighborhoods; water treatment practices increased from 30.3% before the cholera outbreak to 73.9% after the outbreak (p<0.05), and the 2 most common methods used were water purification tablets (66.6%) and bleach (57.7%) (Table 1). Water purification tablets were considered palatable by most respondents (87.7%), and 70.2% reported purchasing them in the past month (Table 2).

**Table 1:** Drinking water sources and treatment before and after cholera outbreak, as reported by survey respondents, Port au Prince, Haiti, 2010*

Source or treatment	Before outbreak			After outbreak	
	No. yes/total no. respondents	Weighted % (95% CI)		No. yes/total no. respondents	Weighted % (95% CI)
Water source					
Piped public kiosk	122/396	32.5 (21.3–43.7)		84/391	21.5 (10.5–32.5)
Piped in house	101/396	26.9 (15.1–38.7)		57/391	15.1 (7.9–22.2)
Private kiosk	129/396	26.8 (18.7–34.9)		203/391	47.6 (36.2–58.9)
Tank filled by truck	11/396	4.4 (0–8.6)		12/391	5.1 (0.8–9.4)
Bladder	3/396	0.6 (0–1.2)		8/391	3.2 (0–8.0)
Other source	7/396	1.3 (0–3.3)		7/391	1.9 (0–3.9)
Treated water (any method)	130/405 (30.3)	30.3 (22.1–38.4)		307/405	73.9 (67.2–80.6)
Method of treatment†					
Water purification tablets	79.119 (66.6)	66.6 (52.8–80.4)		259/301	86.1 (80.2–92.0)
Bleach	76.132 (57.7)	57.7 (47.6–67.8)		174/347	50.1 (36.2–64.1)
Boiling	11/162 (6.8)	6.8 (2.9–10.7)		25/385	6.5 (3.4–9.6)
PuR, Gadyen Dlo, or Dlo Lavi	0	NA		1/333	0.3 (0–0.8)
Other answer	4/160 (2.5)	2.5 (0–5.5)		2/100	2.0 (1.3–3.3)

*CI, confidence interval; NA, not applicable. Sampling weights are according to population size. Before and after data were collected at 1 time point. †Respondents selected >1 method of water purification.

**Table 2:** Access to soap and attitudes toward water purification tablets, Port au Prince, Haiti, December 6−7 and 14−16, 2010*

Access and attitude	No. respondents	Weighted† % (95% CI)
Soap		
Received soap	65	16.5 (3.6–29.4)
Purchased soap	381	95.7 (93.9–97.5)
Had soap at the house at time of survey	355	84.1 (81.3–86.9)
Water purification tablets		
Received in the past month	178	41.5 (29.9–53.1)
Bought in the past month, n = 403	279	70.2 (64.3–76.2)
Know how to use, n = 402	389	97.5 (96.0–99.1)
Perceptions of water purification tablets, n = 387		
Strong taste, unacceptable	25	4.7 (2.4–7.0)
Some taste, acceptable	345	87.7 (83.7–91.6)
No taste	3	0.6 (0–1.2)

*n = 404 except as indicated. CI, confidence interval. †Sampling weights are according to population size.

Among 403 (99.5%) households, ≈60% of samples from stored drinking water tested positive for residual chlorine (range 0–3.5 mg/L). Additionally, during the survey administration, nearly all (94.4%) water storage containers had a cover on them that could reduce the chance of contamination.

Hygienic practices (e.g., handwashing and latrine use) are critical for preventing the spread of diarrheal diseases (*7*–*10*). Active acceptance of these practices and use of soap was high among respondents. Approximately 94.1% reported washing their hands with soap; 84.1% reported having access to soap, 95.7% reported purchasing soap, and 16.5% reported receiving soap from a distribution location since the outbreak started (Table 2). Use of improved latrines was also reported by most respondents (74.0%).

ORS is a lifesaving therapy for diarrheal diseases, including cholera (*11*). Nearly 90% of respondents stated that they knew the method of ORS preparation, although only 76.0% of respondents indicated the correct volume of water needed to prepare an ORS sachet as recommended by the World Health Organization (*12*). One fourth of respondents had ORS in their home when the survey was conducted.

This investigation had several limitations. Because of security restrictions, independent enumerators could not be used. Therefore, some of the enumerators were persons who participated in the implementation of the cholera prevention activities in these communities. Their presence might have biased certain respondent answers. Additionally, sampling was based on available population data from the 2003 census. Migration is likely to have occurred after the earthquake and might have resulted in nonproportional sampling. Finally, sanitation is a sensitive subject within Haitian culture; thus, self-reported access to latrines might be exaggerated. Despite these limitations, the survey provided valuable information reflecting the impact of the public health response to the outbreak and identified areas for improvement.

## Conclusions

Overall, the knowledge of cholera symptoms, prevention, treatment, and modes of transmission indicated that public health messages had been effective. Cholera messaging was successful in promoting behavior changes to address the threat of cholera, especially in increasing acceptance of drinking chlorinated water. Recommendations include additional education campaigns to improve knowledge of correct dosing of water with water purification tablets, ORS preparation, and cholera-prevention methods. Additional follow-up is needed to ensure wide-scale availability of household water treatment products and instruction on proper dosing. Public health officials should take advantage of the substantial interest in and acceptance of chemical water treatment and develop sustainable household water treatment programs. More importantly, a concerted effort should be made to improve the safety of water sources through infrastructure upgrades and improved treatment practices. These upgrades would spur timely sanitary reform and improvements to the public health system of Haiti, which occurred over a century ago in Europe, North America, and most recently in Latin America after the introduction of cholera in the 1990s (*13*,*14*).

## Supplementary Material

Technical Appendix The following pages show a household questionnaire that was pilot tested and administered to 405 households from 27 clusters from resources-limited areas of Cite Soleil, Delmas, Carrefour, and Petion-Ville, Haiti.
