# Supplementary material for: Knowledge, Attitudes, and Practices Related to Treatment and Prevention of Cholera, Haiti, 2010
Source: Emerg Infect Dis. 2011 Nov;17(11):2158–61. doi: 10.3201/eid1711.110818 (PMC3310585; doi:10.3201/eid1711.110818)
Supplement: Technical Appendix — The following pages show a household questionnaire that was pilot tested and administered to 405 households from 27 clusters from resources-limited areas of Cite Soleil, Delmas, Carrefour, and Petion-Ville, Haiti. [file 11-0818-Techapp_9p.pdf]

Article DOI: http://dx.doi.org/10.3201/eid1711.110818

Knowledge, Attitudes, and Practices Related to Treatment and Prevention of Cholera, Haiti, 2010

Technical Appendix

The following pages show a household questionnaire that was pilot tested and administered to 405 households from 27 clusters from resources-limited areas of Cité Soleil, Delmas, Carrefour, and Pétion-Ville, Haiti. The questionnaire was part of a survey conducted during December 6–7 and 14–16, 2010, to assess the effectiveness of interventions implemented to prevent the spread of cholera and to improve specific response activities in these neighborhoods.

ENUMERATOR: ___________________ SUPERVISOR: ___________________
DATE: (day, month, 2010) ___________________ **HOUSEHOLD CODE: ________________**

SURVEY ON KNOWLEDGE, ATTITUDES, AND PRACTICES RELATED TO TREATMENT AND PREVENTION OF CHOLERA, HAITI, 2010

Potopwens, Ayiti

Q#	QUESTION	RESPONSE
**GENERAL**		
**G1**	Name of commune	G1COM<br>**Name of Commune:_____________________**
**G2**	Section Communale	G2SECCOM<br>**Section Communale:________________**
**G3**	Localité	G3LOCAL<br>**Localité**:________________________
**G4**	House Code	G4HHID<br>**House Code :_________________**
**G5**	GPS Coordinates (North)	G5GPSN<br>**GPS Coordinates (North):_______________**
**G6**	GPS Coordinates (West)	G6GPSW<br>**GPS Coordinates (West)**:______________
**DEMOGRAPHIC INFO**		
**I1**	How old are you ?	I1AGE<br>**________(years)**
**I2**	What sex ?	I2SEX<br>**1. Female**<br>**2. Male**
**I3**	How many people live in this house ?	I3HHPPL<br>**________ (people)**
**I4**	Is there a radio that works in this house ?	I4RADIO<br>**1. Yes**<br>**2. No**
**I5**	Is there a TV that works in this house ?	I5TV<br>**1. Yes**<br>**2. No**
**I6**	Is there a cell phone that works in this house ?	I6CELL<br>**1. Yes**<br>**2. No**
**I7**	Can the female chief of household read a sentence in Creole ? ***(If yes, give her a sentence to read in Creole).***	I7READ<br>**1. Reads easily**<br>**2. Reads with some difficulty**<br>**3. Cannot read**<br>**4. N/A, no female head of household home**
**I8**	What's the last level of education completed by the female head of household ?	I8FEDUC<br>**1. Did not go to school**<br>**2.Some primary school**<br>**3. Completed primary school**<br>**4. Some secondary school**<br>**5. Completed secondary school**<br>**6. University / higher education**

DRAFT

ENUMERATOR: ______________________ **HOUSEHOLD CODE: _________________**
SUPERVISOR: _____________________

**WATER – IN THE PAST / BEFORE CHOLERA**		
**W1**	Before the cholera outbreak, what was your principle of drinking water?<br>***(Circle only one response)***	W1PRESOURCE<br>**1 CAMEP/Network**<br>**2 Water in CAMEP fountain**<br>**3 Kiosk**<br>**4 Bladder**<br>**5 Another water vendor**<br>**6 Borehole**<br>**7 Tank filled by truck**<br>**8 Open Well**<br>**9 Sachet**<br>**10 Rainwater**<br>**11 Basin Prive**<br>**12 Other (specify) _________________**
**W2**	Before cholera, did you drink water most of the time directly from the source, or did you do something to it before drinking it ? ***(Circle only one response)***	W2PRESAFE<br>**1. Drank water directly from source *(Go to W4)***<br>**2. Treated water first**
**W3**	What did you do to treat your drinking water ?<br>***(Do not read ; circle all methods mentioned)***	W3PRETRT<br>**1. Boiled water**<br>**2. Jif/Clorox**<br>**3. Aquatabs**<br>**4. PuR**<br>**5. Gadyen Dlo, or Dlo Lavi**<br>**6. Filter: ________________(type of filter)**<br>**7. Solar**<br>**8. Let water settle if murky**<br>**9. Other answer _____________________**
**WATER – NOW – AFTER CHOLERA OUTBREAK**		
**W4**	Since cholera, what is your principle drinking water source ? ***(Circle only one response).***	W4SOURCE<br>**1 CAMEP/Network**<br>**2 Water in CAMEP fountain**<br>**3 Kiosk**<br>**4 Bladder**<br>**5 Another water vendor**<br>**6 Borehole**<br>**7 Tank filled by truck**<br>**8 Open Well**<br>**9 Sachet**<br>**10 Rainwater**<br>**11 Basin Prive**<br>**12 Other (specify**
**W5**	Since cholera, where else do you get drinking water ?<br>***(Circle only one response)***	W5OTHERSOURCE<br>**1 CAMEP/Network**<br>**2 Water in CAMEP fountain**<br>**3 Kiosk**<br>**4 Bladder**<br>**5 Another water vendor**<br>**6 Borehole**<br>**7 Tank filled by truck**<br>**8 Open Well**

DRAFT

		9 Sachet<br>10 Rainwater<br>11 Basin Prive<br>12 Other (specify
**W6**	Since cholera, do you drink your water directly or treat it most of the time?	W6SAFE<br>1. **Drink Directly *(Go to W7)***<br>2. **Treat it *(Go to W6A)***
**W6A**	How do you treat your water ?	W6AHOWTREAT<br>**1. Boiled water**<br>**2. Jif/Clorox**<br>**3. Aquatabs**<br>**4. PuR**<br>**5. Gadyen Dlo, or Dlo Lavi**<br>**6. Filter: _________________(type of filter)**<br>**7. Solar**<br>**8. Let water settle if murky**<br>**9. Other answer**
**W7**	Where do you get water for cooking, bathing, and housework ? ***(Read the list ; circle each response mentioned)***	W7HHWAT<br>**1 CAMEP/Network**<br>**2 Water in CAMEP fountain**<br>**3 Kiosk**<br>**4 Bladder**<br>**5 Another water vendor**<br>**6 Borehole**<br>**7 Tank filled by truck**<br>**8 Open Well**<br>**9 Sachet**<br>**10 Rainwater**<br>**11 Basin Prive**<br>**12 Other (specify**
**SANITATION**		
**S1**	Where do you usually defecate? ***(Circle only one response)***	S1LATRINE<br>**1. Latrine with cement slab**<br>**2. Latrine without cement slab**<br>**3. Trench Latrine**<br>**4. Canal or Open defecation*(Go to K1)***<br>**5. Toilet Mobile *(Go to K1)***<br>**6. Elevated toilet *(Go to K1)***<br>**7. Private toilet**<br>**8. Plastic bag *(Go to K1)***<br>**9. Other __________________ *(Go to K1)***
**S2**	How many households share this latrine ?	S2LATUSE<br><br>**_________ (number of households)**
**S3**	Where is the latrine located ?	S3LATLOCATION<br>**1. In a camp**<br>**2. In a neighborhood**<br>**3. In a public location (school, hospital, etc)**<br>**4. In a private home**<br>**5. Other _____________**
**KNOWLEDGE**		

DRAFT

ENUMERATOR: ______________________ **HOUSEHOLD CODE: _________________**
SUPERVISOR: _____________________

**K1**	Can you tell me the symptoms of cholera ? Symptoms are signs that tell us that someone is sick, for example, coughing, headache, etc. ***(Do not read ; circle each response mentioned and ask what else after each repsons?).***	K1SYMPTOMS<br>**1. Watery diarrhea**<br>**2. Diarrhea**<br>**3. Vomiting**<br>**4. Fever**<br>**5. Dehydration**<br>**6. No appetite**<br>**7. Fatigue**<br>**8. Stomach ache**<br>**9. Other _______________________**<br>**10. Don't know**
**K2**	Can you tell me how people can contract cholera ?<br>***(Do not read ; circle each response mentioned and ask what else ?)***.	K2CONTRACT<br>**1. Drinking contaminated or untreated water**<br>**2. Eating contaminated or undercooked food**<br>**3. Unpleasant odors**<br>**4. Flies**<br>**5. Not washing hands**<br>**6. Contact with someone sick with cholera or someone who died from cholera**<br>**7. Swimming**<br>**8. Other _______________**<br>**9. Unknown**
**K3**	What's the best way for a health worker to share information with you and your neighbors about cholera ? ***(Read the list ; circle more than one response.)***	K3REACH<br>**1. Doctor, nurse, other healthcare worker**<br>**2. SMS**<br>**3. TV**<br>**4. Newspaper**<br>**5. Printed flyers**<br>**6. Public health officials**<br>**Radio**<br>**7. Community meeting**<br>**8. Church**<br>**9. Radio**<br>**10. Community health worker**<br>**11. Truck with megaphone**<br>**12. Other ___________________**
**K4**	Please tell me all of the ways you know to protect yourself against cholera ***(Do not read ; circle all answers mentioned)***	K4PROTECT<br>**1. Boil or treat your water**<br>**2. Use latrines**<br>**3. Wash hands with soap and water**<br>**4. Cook food well**<br>**5. Wash fruits and vegetables**<br>**6. Clean your home with bleach**<br>**7. Other ___________________**
**K5**	If you thought someone in your family had cholera, where would you seek treatment ?	K5SEEKCARE<br>**1. Treat at home (GO to K5A)**<br>**2. Go to health facility(GO to K5B)**
**K5A**	Why would you not seek care away from home ?	K5AWHYNOTSEEK<br>**1. Waiting to see if ORS works**<br>**2. No Security**<br>**3. Transportation not available**<br>**4. No money to pay for treatment**

DRAFT

		**5. Transportation not affordable**<br>**6. Because I drink ORS**<br>**7. I will try traditional healers first**<br>**8. No room at the treatment center (too much waiting)**<br>**9. Other_________________**
**K5B**	How long would you wait to go to the health center, hospital, or CTC ?<br>What is the largest you would wait to go to the hospital/ health center/CTC ?	K5BWHENSEEK<br>**1. Less than 2 hours**<br>**2. More than 2 hours**<br>**3. After one day**<br>**4. Don't know**
**K6**	Has anyone in your home had cholera ?	K6CHOLERA<br>**1. Yes**<br>**2. No *(Go to DS1)***
**K7**	Did the sick person take ORS or sugar salt solution?	K7ORSTAKE<br>**1.Yes**<br>**2. No**
**K8**	Where did the sick person seek treatment ?<br>***(Circle each response mentioned).***	K8WHERE<br>**1. Health center, clinic, hospital**<br>**2. CTC**<br>**3. Dispensary, pharmacy**<br>**4. Traditional healer : houngan, bòkò,**<br>**5. Other______________________**<br>**6. Did not seek treatment outside of the house**<br>**7. Don't know**
**DISTRIBUTION - SOAP**		
**DS1**	In the past month, did you receive (for free or during a distribution) hand soap ? ***If yes***, how many bars of hand soap ?	DS1GOTSOAP<br>**1. Yes, _________ (bars of soap)**<br>**2. No**
**DS2**	Have you purchased any hand soap since the cholera epidemic started ?	DS2BUYSOAP<br>**1. Yes**<br>**2. No**
**DS3**	Do you have handsoap or powered soap now ?	DS3HANDSOAPNOW<br>**1. Yes**<br>**2. No**
**DS4**	Do you use saop or detergent when you wash your hands ?	DS4HANDSOAPCONFIRM<br>**1.Always**<br>**2..Sometimes**<br>**3.Never**
**DISTRIBUTION – ORS**		
**DO1**	Do you have any ORS now home?	DO1ORSNOW<br>1. **Yes**<br>2. **No**
**DO2**	Do you know how to use/prepare ORS? ***(Circle only one answer).***	DO2INSTORS<br>**1. Yes**<br>**2. No (Go to DO5)**<br>**3. Don't know (Go to DO5)**
**DO3**	How to prepare ORS, how much water do you mix with one sachet of ORS ?<br>***(Do not read ; circle each response mentioned.)***<br>***Choose one***	WaterORS1 **Small Coke Bottles**<br>WaterORS2 **Large Coke Bottles**<br>WaterORS4 **Other ______________________**

DRAFT

ENUMERATOR: ______________________ **HOUSEHOLD CODE: _________________**

SUPERVISOR: _____________________

**DO4**	After you finish preparing a sugar-salt solution or ORS, how long can you keep it until it goes bad ?	DO4HOWLONG<br>**1. Have to use it right away – within an hour**<br>**2. Within 5 hours**<br>**3. Within 24 hours**<br>**4. More than one day**<br>**5. Don't know**
**DO5**	If you don't have ORS what else you can use ?	DO5ORSELSE<br>**1.Sugar-salt solution**<br>**2.Water**<br>**3.Juce**<br>**4.Other**
**DISTRIBISYON – TRETMAN DLO**		
**DT1**	Have you heard about any of these products :<br>(***Read each choice and circle responses.)***	DT1DLOLAVIDIST<br>**Dlo Lavi**<br>**1. Yes**<br>**2. No**<br>DT1AQUATABDIST<br>**Aquatabs**<br>**1. Yes**<br>**2. No**<br>DT1GADYENDLODIST<br>**Gadyen Dlo**<br>**1. Yes**<br>**2. No**<br>DT1JIF/CLOROXDIST<br>**Jif/Clorox**<br>**1. Yes**<br>**2. No**
**DISTRIBISYON - AQUATABS**		
**DA1**	In the past month did you receive Aquatabs ?<br>***If so***, How many tabs ?	DA1GOTAT<br>**1. Yes, __________ (tabs)**<br>**2. No**
**DA2**	In the past month have you bought any Aquatabs ?	DA2BOUGHTAQUATAB<br>**1. Yes**<br>**2. No**
**DA3**	Do you know how to use Aquatabs ?	DA3INSTAT<br>**1. Yes**<br>**2. No *(Go to DA8)***<br>**3. Don't know *(Go to DA8)***
**DA4**	*How many pills of Aquatabs do you use for 5 gallons of water?*	DA4NUMUSE<br>**Number of Aquatabs :_______**
**DA5**	*What size of Aquatabs do you use ?*	DA5SIZEUSED<br>**Size of Aquatabs :________________**
**DA6**	*How much water do you use to treat water with Aquatabs every day?*	DA6LITERSUSED<br>**Liters of Water :________________**
**DA7**	After you add Aquatabs to water, can you drink immediately or should you wait before drinking?	DA7ATWAIT<br>**1. Drink right away *(Go to DA8)***<br>**2. Let it sit *(Go to DA7A)***<br>**3. Don't know *(Go to DA8)***
**DA7A**	How long should you let it sit ?	DA7AHOWLONGSIT<br>**Mintues :_______________**
**DA8**	If you had them, would you use Aquatabs to treat your drinking water ?	DA8ATDRWAT<br>**1. Yes**

DRAFT

		**2. No**
**DA9**	Have you ever tasted water with Aquatabs ?	DA9WTAERAQUATABS<br>**1. Yes**<br>**2. No (Go to DA10)**
**DA9A**	What do you think of the taste of water treated with Aquatabs ? ***(Circle just one response)***	DA9ATTASTE<br>**1. Strong taste, I don't like it**<br>**2. Some taste, but it's fine**<br>**3. No taste**<br>**4.Never taste it**
**DA10**	Do you think the water from the pipe (CAMEP) is safe to drink ?	DA10CAMEPSAFE<br>**1. Yes**<br>**2. No**<br>**3. Don't Know**
**DA11**	Do you think the water from a kiosk (Eau Miracle, etc) is safe to drink ?	DA11KIOSKSAFE<br>**1. Yes**<br>**2. No**<br>**3. Don't Know**
**DA12**	Do you think the water from a bladder or a tank is safe to drink ?	DA12BLADDERSAFE<br>**1. Yes**<br>**2. No**<br>**3. Don't Know**
**DA13**	Do you think the water from a borehole is safe to drink ?	DA13BOREHOLESAFE<br>**1. Yes**<br>**2. No**<br>**3. Don't Know**
**DA14**	Which source of water do you think is safe to drink?<br>***(Do not read ; circle each response mentioned.)***	DA14WATERSAFE<br>**1 CAMEP/Network**<br>**2 Water in CAMEP fountain**<br>**3 Kiosk**<br>**4 Bladder**<br>**5 Another water vendor**<br>**6 Borehole**<br>**7 Tank filled by truck**<br>**8 Open Well**<br>**9 Sachet**<br>**10 Rainwater**<br>**11 Basin Prive**<br>**12 Other (specify**
**OBSERVATION**		
**O1**	Can you show me how you wash your hands ? (Observe how the person washes the hands and circle the answers that correspond to the person does)	O1HANDWASH<br>**1. Water only**<br>**2. Soap and water**<br>**3. Powdered or liquid detergent and water**<br>**4. Ashes and water**<br>**5. Dirt/sand/mud and water**<br>**6. Don't wash hands**<br>**7. Cannot demonstrate, no soap and/or water in home**<br>**8. Other ____________________**
**O2**	What source did this water come from ?<br>***(Circle only one response)***	O2WATSOURCE<br>**1 CAMEP/Network**<br>**2 Water in CAMEP fountain**

DRAFT

		**3 Kiosk**<br>**4 Bladder**<br>**5 Another water vendor**<br>**6 Borehole**<br>**7 Tank filled by truck**<br>**8 Open Well**<br>**9 Sachet**<br>**10 Rainwater**<br>**11 Basin Prive**<br>**12 Other (specify**
**O3**	Is the water container covered ?	O3WATCOV<br>**1. Yes**<br>**2. No**
**O4**	Was this water treated with Aquatabs, Jif, or Clorox?	O4WATTRT<br>**1. Yes**<br>**2. No**
**O5**	How long ago was it treated? ***(Si tan plis yon jouneen, makè 24+.)***	O5WHENTRT<br><br>**_______(hours)**
**O6**	Test and measure the chlorine level in the stored water ***(Make X, si-l pa stoke dlo oubyen refize ; Makè 0 si pa koulè dlo-a pa chanje di tou).***	O6CHLORINE<br><br>**_____ mg/l**

DRAFT
